# Transforming a well into a chip: A modular 3D-printed microfluidic chip

**DOI:** 10.1063/5.0039366

**Published:** 2021-04-28

**Authors:** Rossana Rauti, Adi Ess, Baptiste Le Roi, Yevgeniy Kreinin, Mark Epshtein, Netanel Korin, Ben M. Maoz

**Affiliations:** 1 Department of Biomedical Engineering, Tel Aviv University, Tel Aviv 6997801, Israel; 2 Sagol School of Neuroscience, Tel Aviv University, Tel Aviv 6997801, Israel; 3 Department of Biomedical Engineering, Technion Israel Institute of Technology, Haifa 32000, Israel; 4 The Center for Nanoscience and Nanotechnology, Tel Aviv University, Tel Aviv 6997801, Israel

## Abstract

Organ-on-a-Chip platforms provide rich opportunities to observe interactions between different cell types under *in vivo*-like conditions, i.e., in the presence of flow. Yet, the costs and know-how required for the fabrication and implementation of these platforms restrict their accessibility. This study introduces and demonstrates a novel Insert-Chip: a microfluidic device that provides the functionality of an Organ-on-a-Chip platform, namely, the capacity to co-culture cells, expose them to flow, and observe their interactions—yet can easily be integrated into standard culture systems (e.g., well plates or multi-electrode arrays). The device is produced using stereolithograpy 3D printing and is user-friendly and reusable. Moreover, its design features overcome some of the measurement and imaging challenges characterizing standard Organ-on-a-Chip platforms. We have co-cultured endothelial and epithelial cells under flow conditions to demonstrate the functionality of the device. Overall, this novel microfluidic device is a promising platform for the investigation of biological functions, cell–cell interactions, and response to therapeutics.

## INTRODUCTION

The development of *in vitro* models that recapitulate *in vivo* features is essential for elucidating human physiology and disease mechanisms, as well as for drug discovery.^1–5^ As human physiology is highly complex, such *in vitro* models should ideally take many parameters into account, including the following: cellular microenvironment,^6,7^ cell–cell communication,^8,9^ organ–organ interactions,^4,10,11^ and mechanical aspects such as hydrodynamic and shear stress, which are critical for the development of cellular functionality.^12,13^ In recent years, several *in vitro* modeling platforms have been developed with the capacity to capture many of these features.^4,14–16^ These platforms include Transwell (TW) cell culture inserts, which enable cells to be co-cultured over a membrane;^17–19^ microfluidic devices (Organs-on-a-Chip), which allow for both co-culturing and the application of flow and other mechanical forces;^20^ organoids, which mimic 3D tissue structure; and other 3D-systems that recreate a 3D microenvironment.^4,21,22^

Though these platforms constitute significant advancements toward faithfully recapitulating *in vivo* environments, each has certain shortcomings that hinder its universal application. In particular, as yet, no one system fulfills all of the following criteria: modular, low cost, easy to use, applicable to high-throughput experiments, captures cell–cell interactions, capable of inducing flow, and compatible with high-magnification imaging procedures.

Here, we describe the establishment of a system that brings us closer to achieving this “ideal” by combining the strengths of two popular platforms, namely, TWs and the Organs-on-a-Chip, while overcoming some of their limitations.

TW inserts are commercially available in a range of size, easy to use, and can be used as a high-throughput tool.^23^ Yet, TWs are considered to be “static” models, as they do not have the capacity to induce flow, a crucial feature for models of vasculature and epithelial tissues.^24–26^ The Organs-on-a-Chip, in turn, enables the application of controlled flow and can provide insight regarding organ–organ interactions;^10,11,27,28^ however, Organ-on-a-Chip systems are not modular, and their fabrication and implementation typically require a great deal of time and know-how.^29^ Moreover, most chips are made of polydimethylsiloxane (PDMS), which adsorbs hydrophobic compounds, limiting the platform's applicability to drug testing. An additional shortcoming, shared by both TWs and Organ-on-a-Chip systems, is the substantial difficulty in using high-resolution microscopy to investigate cell dynamics, owing to the large working distance needed for visualizing the cells.

**FIG. 1.**
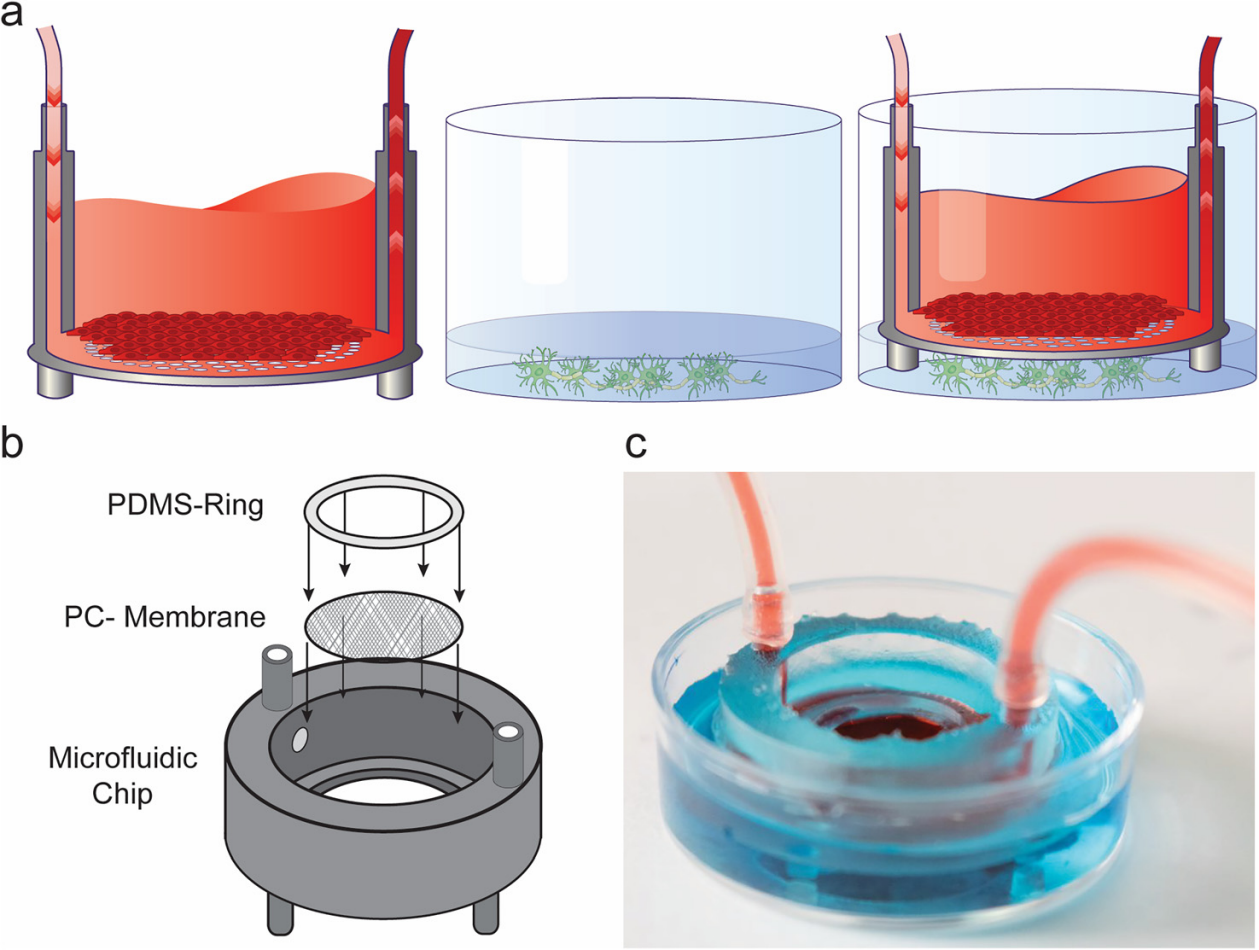
Insert-Chip design. (a) Schematic of the experimental design: The Insert-Chip can be integrated with any standard culture platform. In the schematic, endothelial cells are grown on the top of the porous membrane inside the Insert-Chip, while neurons are grown on the bottom of a well-plate; the Insert-Chip and the well-plate are then integrated together. (b) Exploded view of the Insert-Chip showing the three different components of the platform: the 3D-printed base, a porous PC membrane, and the PDMS ring. (c) Photograph of the assembled Insert-Chip integrated in a petri dish, with two different colored solutions, one inside the chip and the other on the bottom of the plate.

**FIG. 2.**
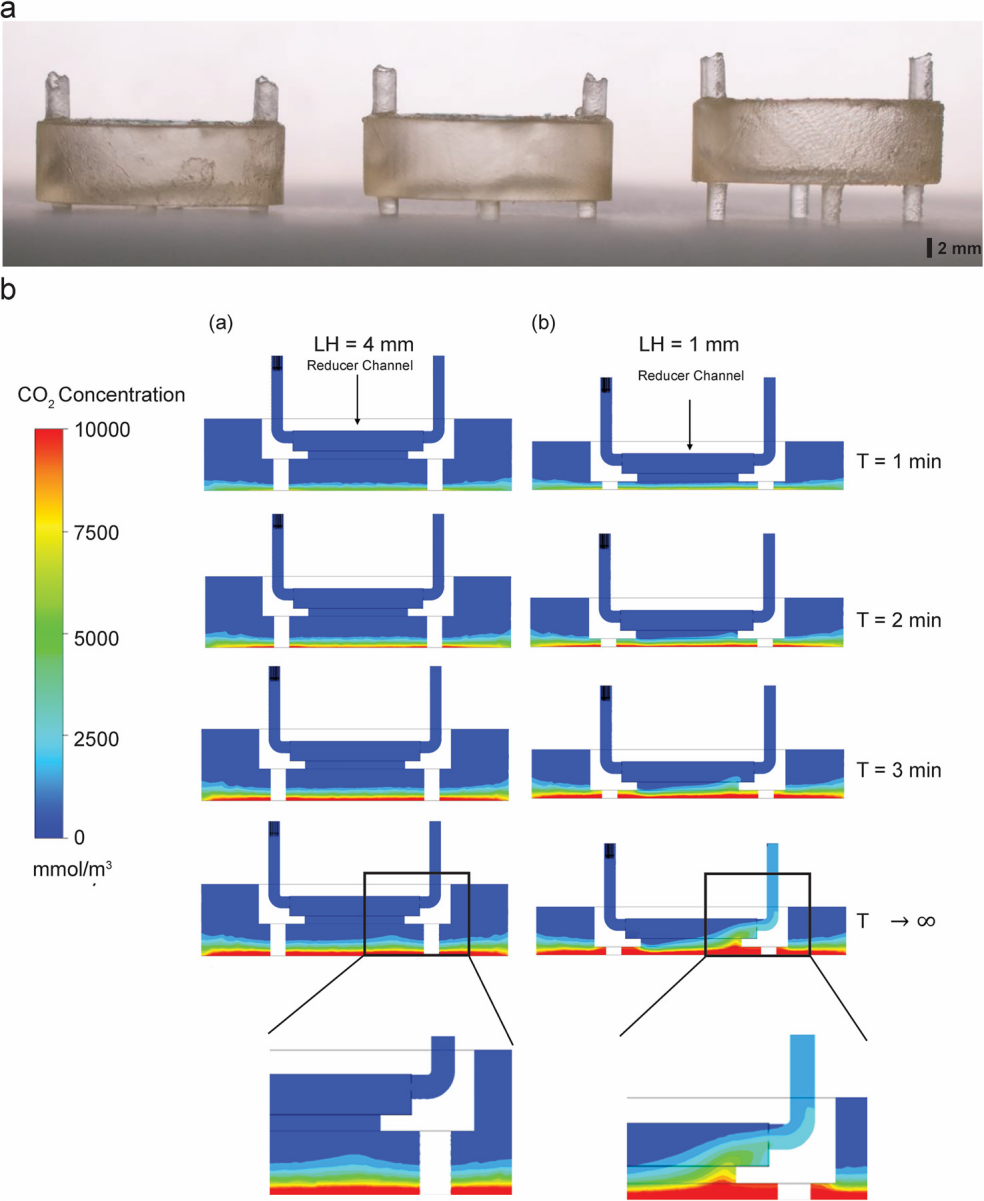
Modularity of the Insert-Chip. (a) 3D-printed Insert-Chip with different leg heights (LH; 1 mm, 2 mm, and 4 mm, respectively). (b) Time series of the diffusion simulations results (a) cross section view of the reduced chip with 4 mm (a) and 1 mm (b) LH showing CO_2_ accumulation at the bottom of the container. In LH = 4 mm it reaches halfway to the reducer channel, and does not extend into the flow even at T → ∞, while in LH = 1 mm CO_2_ reaches the reducer channel at T = 2 min and eventually extend into the outlet tube at T → ∞ (note the enlarged pictures of the bottom compartment).

Several groups have tried to combine different *in vitro* modeling approaches to overcome the challenges outlined above. Sip *et al.*,^30^ for example, developed a TW with flow, which uses soft-lithography to produce PDMS microchannels which are attached to 6-well TW holders. However, the platform they proposed has several key shortcomings; specifically, it requires complex manufacturing procedures such as soft-lithography, it is not versatile (limited to six well plate), has a fixed distance between the bottom of the well and the membrane (i.e., the inserted component containing cultured cells), and does not provide the capacity to image membranes at high magnifications. To capture the benefits of TW inserts and Organs-on-a-Chip, while overcoming their individual and shared limitations, we used new fabrication tools (3D printing) to develop an easy-to-use, customizable, microfluidic chip that, similarly to a TW, can be inserted into any standard culture platform. This cylindrical *Insert-Chip* (Figs. 1 and 2) is 3D-printed from clear dental resin, with a single porous membrane-on which cells can be cultured-positioned near its base.

**FIG. 3.**
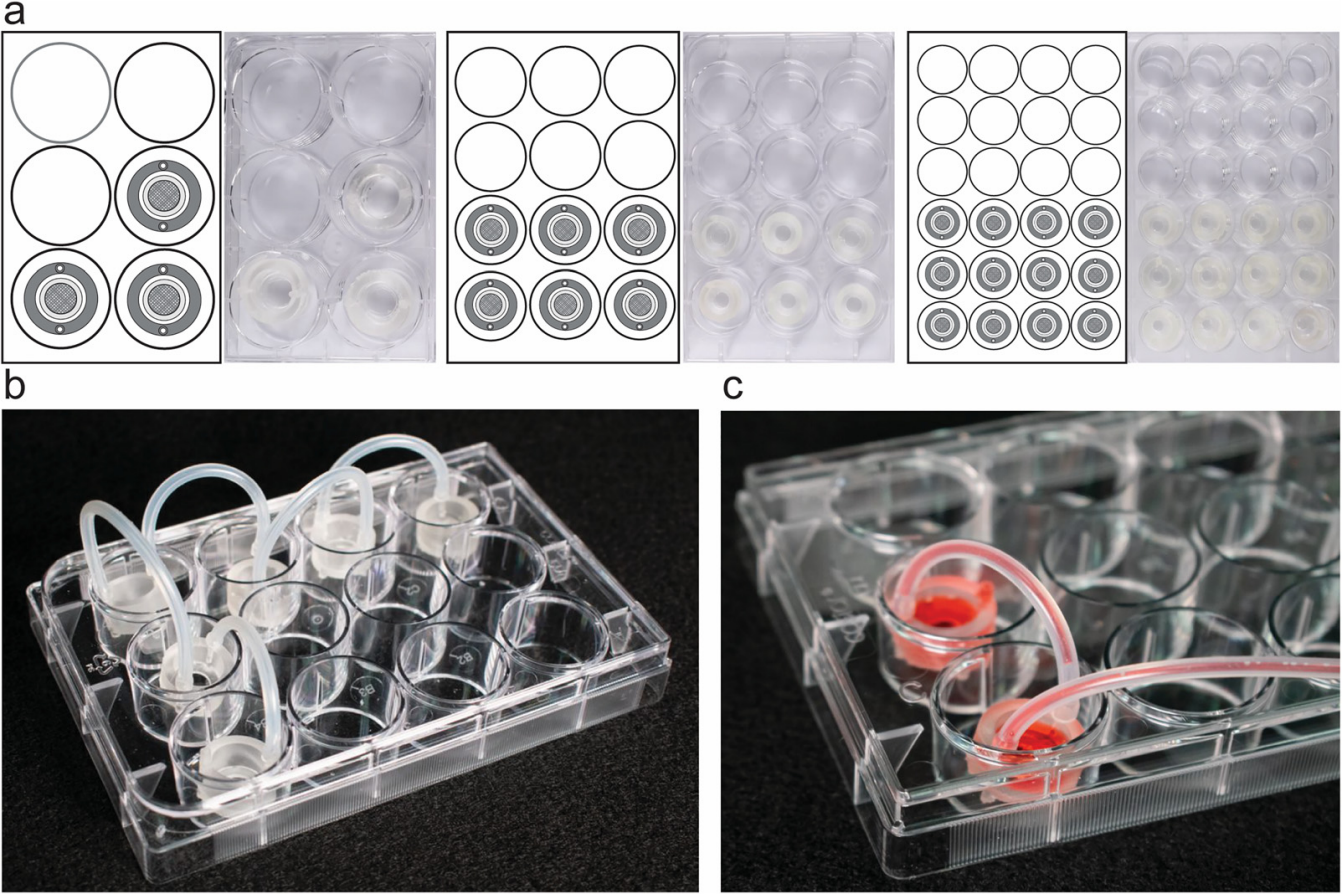
Versatility of the Insert-Chip. (a) Insert-Chips fabricated in different sizes in order to be integrated with commercially available 6, 12, and 24 well-plates. (b) 6 Insert-Chips integrated in a 12-well plate and linearly connected to one another in order to simulate multi-organ-chip platforms. (c) Magnification of 2 Insert-Chips connected under flow.

The membrane is situated within the chip with the support of a PDMS ring. The chip contains inlet and outlet openings that can be used to connect the chip to a flow system. The Insert-Chip stands on four short legs (1–2 mm long) and thus can stand alone in either a well plate or multi-electrode array (MEA) environment, above a cell culture surface, thereby enabling the cells in that environment to interact with the cells in the chip. The chip is re-usable (after simply cleaning and sterilizing it with ethanol and UV lamp), allows for advanced imaging and sensing, and can be used in high-throughput platforms, while providing the capacity to assess organ–organ interactions (Figs. 2 and 3).

As a proof of concept, we 3D-printed Insert-Chips in different sizes to demonstrate their modularity and adaptability to standard cell culture platforms commonly used in a lab. In addition, we carried out experiments in which we cultured barrier tissue cells (either endothelial or epithelial cells) on top of the Insert-Chip membrane; we used these experiments to demonstrate the capacity to induce controlled flow in the Insert-Chip and to image cells with high-resolution confocal microscopy. Furthermore, we demonstrated how the chip can be integrated into conventional culturing platforms, while providing the capacity to co-culture cell populations in the presence of flow. To this end, we inserted an Insert-Chip cultured with endothelial cells into an MEA containing parenchymal cells (neurons and astrocytes). We demonstrated endothelial and neuronal cell functionality via simultaneous barrier and electrophysiological measurements. Finally, experiments with modified versions of the Insert-Chip hint at additional design features that might further improve the chip's efficiency or suitability for specific types of experiments.

The promising results of our experiments highlight the potential of the Insert-Chip as a straightforward yet advanced *in vitro* modeling platform that can benefit both academic and pharmaceutical labs.

## RESULTS AND DISCUSSION

### Insert-Chip design

The goal of this work was to develop a modular, inexpensive, and user-friendly chip that exposes cultured cells to a controllable flow and that supports cell–cell interactions and co-cultures. Most importantly, the Insert-Chip can be integrated into a variety of standard well plate cell culture platforms [Fig. 1(a)], including MEA platforms.

Broadly, each Insert-Chip contains a cell culture chamber with an external diameter customizable to up to 25 mm and an inner diameter of 17 mm, with capacity of up to 2 ml of cellular medium. Inlet and outlet channels on the upper part of the chip enable the chamber to be connected to a controlled flow system [see Figs. 1(b) and 1(c)]; the inlet and outlet channels are 5 mm high, with external and internal diameters of 2.5 mm and 1.5 mm, respectively. The bottom part of the chip includes four small, modular legs, which enable the device to be self-standing, while providing visual access to the membrane (e.g., for continuous microscopic visualization of cell growth).

**FIG. 4.**
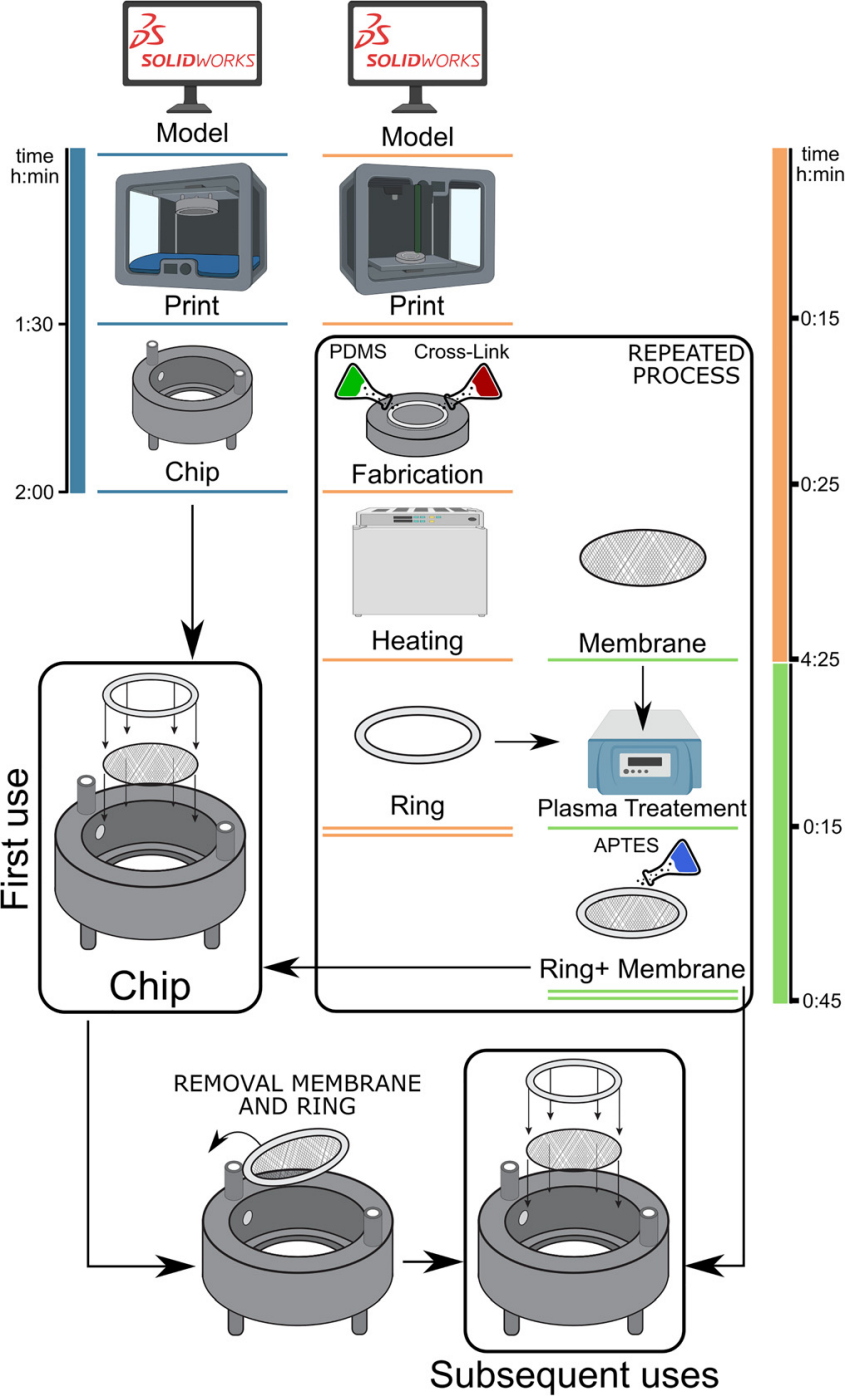
Insert-Chip fabrication process. Schematic time-line representation of the Insert-chip and the ring fabrication followed by the chip assembly (created with Biorender.com). To note that once the Insert-Chip was fabricated, it can be easily reused only disassembling the ring with the membrane.

The Insert-Chip has several key design aspects that overcome the current limitations of Organs-on-a-Chip, by leveraging the strengths of static TW inserts: (1) compatibility: the Insert-Chip is a stand-alone platform that can be integrated into almost any standard culturing platform (6, 12, or 24-well plate or MEA substrate) (Figs. 1 and 3), and, in doing so, transform it into an Organ-on-a-Chip system. This feature enables cells to be cultured without undergoing special optimization procedures (in contrast to regular Organs-on-a-Chip). Indeed, cells with different stages of maturation and functionality can be cultured separately on the well plate and on the Insert-Chip. Once the cultures are ready for the experiment, the Insert-Chip can be added. To achieve straightforward integration into standard culture platforms, we designed the Insert-Chip to be self-supported on four short legs (approximately 1–2 mm in length) with the membrane positioned below the cell culture surface (Fig. 1) in any orientation desi (2) co-culture: A key feature of Organs-on-a-Chip is the capacity to accommodate cell–cell interaction and diffusion between compartments (Fig. 2 and supplementary material Fig. 1). To achieve this property, we designed the Insert-Chip to have a porous membrane that allows the possibility to create gradient and diffusion between different cell cultures. Furthermore, it allows up to three different cell types to be cultured and potentially to interact within a single experiment {on top of the membrane, on the bottom of the membrane, and on the bottom of the well, into which the Insert-Chip is inserted [Figs. 1(a) and S1]}. (3) Flow and shear stress: The Insert-Chip was designed in a configuration that enables different flow configurations (Figs. S2 and S3) and shear forces [Fig. 2(b)] to be induced on the cells. It is important to note that, *in vivo*, epithelial and endothelial cells are constantly subjected to flow, and it is essential for *in vitro* platforms to recapitulate these conditions. (4) Fabrication: The Insert-Chip was designed so that it can be fabricated by a regular 3D printer, using transparent materials such as a PC membrane and clear dental resin, which allow for real-time observations of cell morphology. Moreover, the Insert-Chip is designed in such a way that the membrane can be easily disassembled, enabling cells to be imaged at high resolution. Notably, this feature enables the Insert-Chip to be reused (Fig. 4), making it cost-efficient.

### Insert-Chip fabrication

Most Organs-on-a-Chip or microfluidic devices are fabricated from PDMS, which is biocompatible, transparent, and has good gas permeability. However, a major limitation of PDMS is its hydrophobicity, which causes substantial absorption of hydrophilic materials. Moreover, in some cases, chip fabrication requires specific know-how and facilities. To overcome these challenges, we used stereolithograpy 3D printing for fabricating the Insert-Chip. The use of 3D-printing enables the design of the desired platform to be quickly modified, and it reduces the need for multi-step fabrication needed in “standard” Organs-on-a-Chip. Furthermore, the use of 3D-printing reduces the fabrication time of the Organ-on-a-Chip from several days to a few hours (Fig. 4), as well as the possibility to use not-absorbing materials. The Insert-Chip is made only from three parts [Fig. 1(b)]: base, membrane, and sealing ring. The base is fully made with a 3D printer (see Materials and Methods section). The membrane can be versatile, i.e., there are no restrictions on what material can be used. In this study, we used porous PC for the membrane (0.4 *μ*m pore size) [Fig. 1(b)]. The membrane is interfaced to the Insert-Chip with a ring (16 mm external and 13 mm inner diameter) made of PDMS, previously fabricated in a specific 3D-printed mold (see Materials and Methods for details). To ensure complete adherence between the membrane and the sealing ring, we used plasma and APTES, as previously described;^32^ this process ensures long-term stability,^32^ which is crucial for reusing the Insert-Chip and for allowing diffusion between the two compartments, as demonstrated in Fig. 1(c) and Movie S3, using different color solutions.

### Insert-Chip modularity and compatibility with standard *in vitro* platforms

An important feature of our Insert-Chip is the fact that “one-design fits all,” i.e., the chip is modular and can be integrated with existing platforms. One of the strengths of the standard dual-channel Organ-on-a-Chip platform is that it provides the capacity to observe cell–cell interactions. With the Insert-Chip, cell–cell interactions can take place between the cells plated on the device membrane and the cells cultured in the well into which the device is inserted. The characteristics of these interactions are mainly determined by the flow rate, pore size of the membrane, and the distance between the two cell populations (the distance between the membrane and the bottom of the plate). As our Insert-Chip is fabricated via 3D printing, all these parameters can be adjusted in accordance with experimental requirements. For example, Fig. 2(a) shows an example in which the length of the Insert-Chip's legs is adjusted to change the distance (height) between the membrane and the bottom plate. This versatility is especially important for controlling the diffusion, material gradient, and shear forces between the upper and lower compartments.^30^ For proof of principle, we fabricated Insert-Chips with three different heights [Fig. 2(a)], 1 mm, 2 mm, and 4 mm and we simulated the diffusion of CO_2_ in the Insert-Chip with 1 mm and 4 mm legs-height (LH) [Fig. 2(b)]. The diffusion simulations show that the general influence of the chip LH is to control the relative influence of convective vs the purely diffusive mass transport with increased LH. This trend is demonstrated by the stable diffusive front in the 4 mm LH configuration where the reducer flow chamber is relatively far from the CO_2_ producing cells at the bottom and does not induce a significant convective transport in the container. Thus, even at infinite time, there is no CO_2_ in the reducer, and the concentration at the bottom will continue to rise unhindered until saturation [Fig. 2(b) and Movie S1]. On the other hand, when the reducer is closer to the source of the CO_2_ the entire distribution map is skewed toward the outlet resulting eventually in removal of mass through the outlet when the system reaches a steady-state [Fig. 2(b) and Movies S1 and S2]. This trend will vary in intensity in different system configurations but will always be present due to the low pressure created by the flow, even when the membrane will be in place. The capacity to insert the chip into almost any standard *in vitro* platform [e.g., 6, 12, and 24 well plates, Fig. 3(a)] is a key benefit for biomedical experiments, as this feature contributes toward cost-efficiency, reduces the need for customized equipment, and enables high-throughput systems (e.g., 24-well plates) to be used as “dual-compartment Organs-on-a-Chip.”

Moreover, when multiple Insert-Chips are placed next to each other [Fig. 3(a)], it is possible to link them together [Figs. 3(b) and 3(c)] and thus to create multi-organ-chip systems. Furthermore, two different cells types can be cultured on each side of the membrane and placed in contact with cells grown in another support, such as a well-plate, which creates a tri-culture system as shown in Fig. S1.

This feature can contribute substantially to the study of human physiology and pharmacokinetics and pharmacodynamics, for which organ–organ interactions are crucial, yet highly challenging to mimic *in vitro.*^28,40,41^

### Endothelial and epithelial barriers

**FIG. 5.**
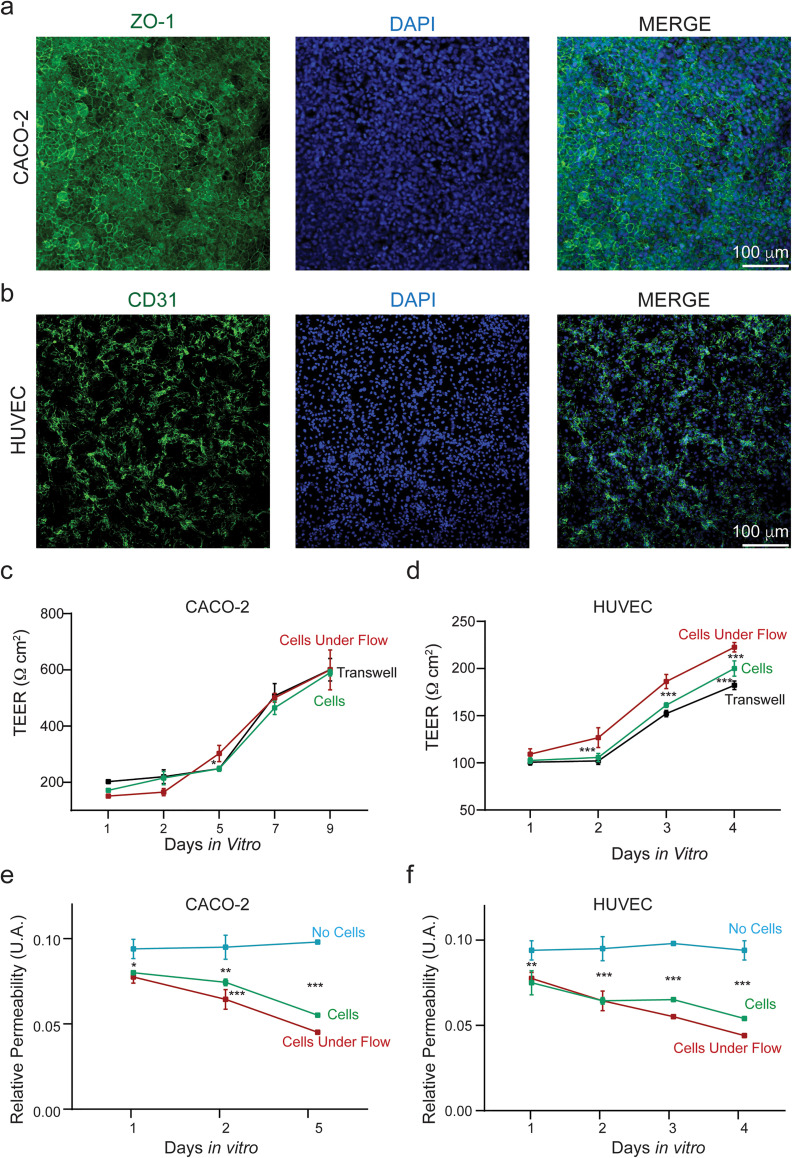
Epithelial and endothelial barrier grown on the Insert-Chip. (a) Confocal reconstructions of epithelial (Caco-2) cells immunostained for ZO-1 (green) and nuclei (DAPI); (b) confocal reconstructions of endothelial (HUVEC) cells immunostained for CD31 (green) and nuclei (DAPI); (c) plot showing pooled TEER values of Caco-2 and (d) HUVEC cultured on the Insert-Chip with and without flow and on Transwells. (e) Relative Permeability Values of Caco-2 and (f) HUVEC cells measured as leakage of FITC-dextran from the upper to the bottom compartment of the Insert-Chip.

We sought to demonstrate the use of the Insert-Chip as a modular “Epithelium-on-a-Chip” (Caco-2 cells) or “Endothelium-on-a-Chip” (HUVEC) [Figs. 5(a) and 5(b)]. We chose these cell types because all parenchymal tissues interact with barrier tissues, and it is known that these tissues show better properties under flow,^42,43^ and the capacity to induce controlled flow is one of the strengths of the system.

We monitored cell growth and barrier development over 4 and 9 days (from 1 to 4 or from 1 to 9 DIV), until the Caco-2 cells and HUVEC formed complete confluent monolayers [Figs. 5(c) and 5(d), respectively]. Once the cells showed confluent monolayers, barrier function was further tested via immunocytochemistry [Fig. 5(a) and 5(b)], demonstrating a continuous distribution of tight junctions in both cellular types. In addition, both TEER and permeability measurements were used to assess barrier function over the course of the observation period [Figs. 5(c)–5(f)].

Both methods give complementary information on the barrier properties, as TEER provides a quick, noninvasive, and real-time indication of barrier properties;^44,45^ while fluorescence assays can provide information on how the permeability changes with the molecular weight, it is important to note that the design of the Insert-Chip allows for the use of commercial TEER systems. TEER measurements were used to compare our Insert-Chip system to the ones measured on commercially available Transwells.

No significant differences were found in Caco-2 cells cultured under flow (from 151.2 ± 6.2 Ω cm^2^ to 600.0 ± 70.7 Ω cm^2^) compared to the ones grown without flow (from 171.2 ± 6.2 Ω cm^2^ to 590.0± 11.5 Ω cm^2^) or on Transwells [from 202.5 ± 5.0 Ω cm^2^ to 600.5± 40.0 Ω cm^2^, Fig. 5(c)]. Conversely, when comparing HUVEC cells, significant differences were found between cells grown in the Insert-Chip under flow (from 109.2 ± 5.6 Ω cm^2^ to 222.5 ± 5.0 Ω cm^2^) to the ones without flow (from 102.5 ± 2.8 Ω cm^2^ to 200.0 ± 8.1 Ω cm^2^) or on Transwells [from 100.7 ± 2.9 Ω cm^2^ to 182.2 ± 4.5 Ω cm^2^; Fig. 5(d)].

To validate our platform, permeability measurements were done without cells and cells that were cultured with and without flow.

This was done by quantifying the rate at which water soluble fluorescein isothiocyanate (FITC)-dextran was transported across the endothelium and epithelium to the bottom compartment of the Insert-Chip upon addition at the upper one [Figs. 5(e) and 5(f)]. A significant decrease in terms of absorption measurement after 1, 2, and 5 days for the Caco-2 cells [Fig. 5(e)] and after 1, 2, 3 and 4 days for the HUVEC cells [Fig. 5(f)] (in static and under flow condition) confirmed the establishment of cellular barriers, compared to Insert-Chip without cells. Or in other words, it can be seen that cells created a barrier layer and that flow induction enhanced the barrier properties.

### High-resolution imaging capabilities

**FIG. 6.**
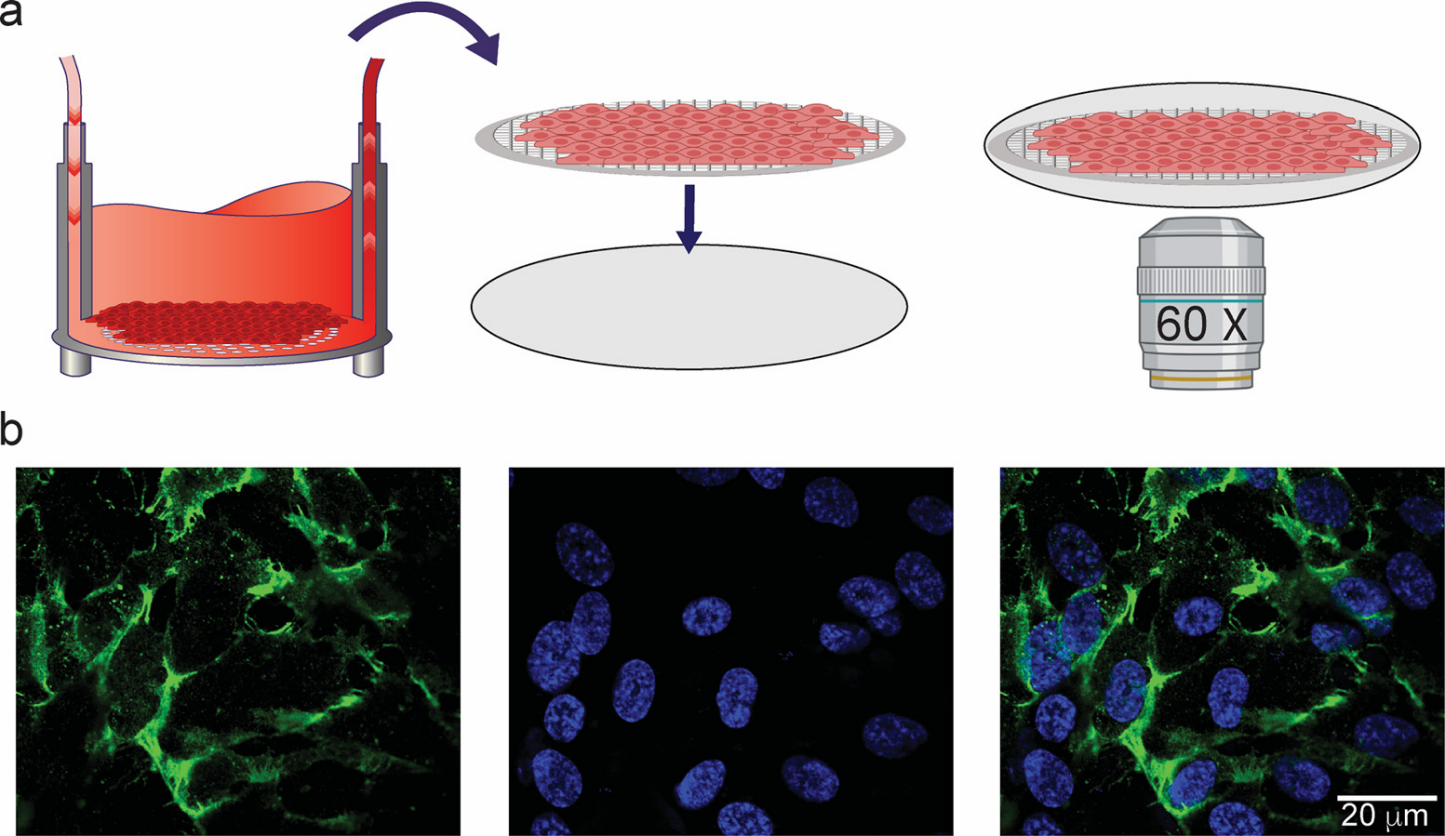
High-resolution imaging of cells cultured in the Insert-Chip. (a) Schematic design of easy removal of the porous membrane containing cultured endothelial cells, in order to perform high-resolution confocal imaging. (b) Confocal reconstructions at 60× magnification of HUVEC cells cultured on the porous membrane and stained for CD-31 (green) and DAPI (blue).

High-resolution imaging is an indispensable tool for studying the structure and the dynamics of cells. Unfortunately, it is highly challenging to do high-magnification imaging with standard dual-channel Organs-on-a-Chip, as the typical working distance of 40×, 60× objectives is 170–200 *μ*m, and the distance of the membrane where cells are cultured from the bottom of the Chip is usually above 300 *μ*m. To overcome this challenge, we designed the Insert-Chip in such way that the membrane can be easily removed from the chip (Fig. 6) after the culture period, due to the presence of the PDMS ring, by simply using a tweezer.

Once the membrane is removed, it can be placed on a glass coverslip, and standard immunocytochemistry can be performed on the membrane, which can be mounted onto a glass slide for high-magnification imaging [Fig. 6(a)]. As shown in Fig. 6(b), high magnification (60× oil objective) of HUVEC, stained for CD-31 protein (in green) and DAPI (blue) for the nuclei, enables cell junctions to be better identified and investigated.

### Chip reducer and shear force application

**FIG. 7.**
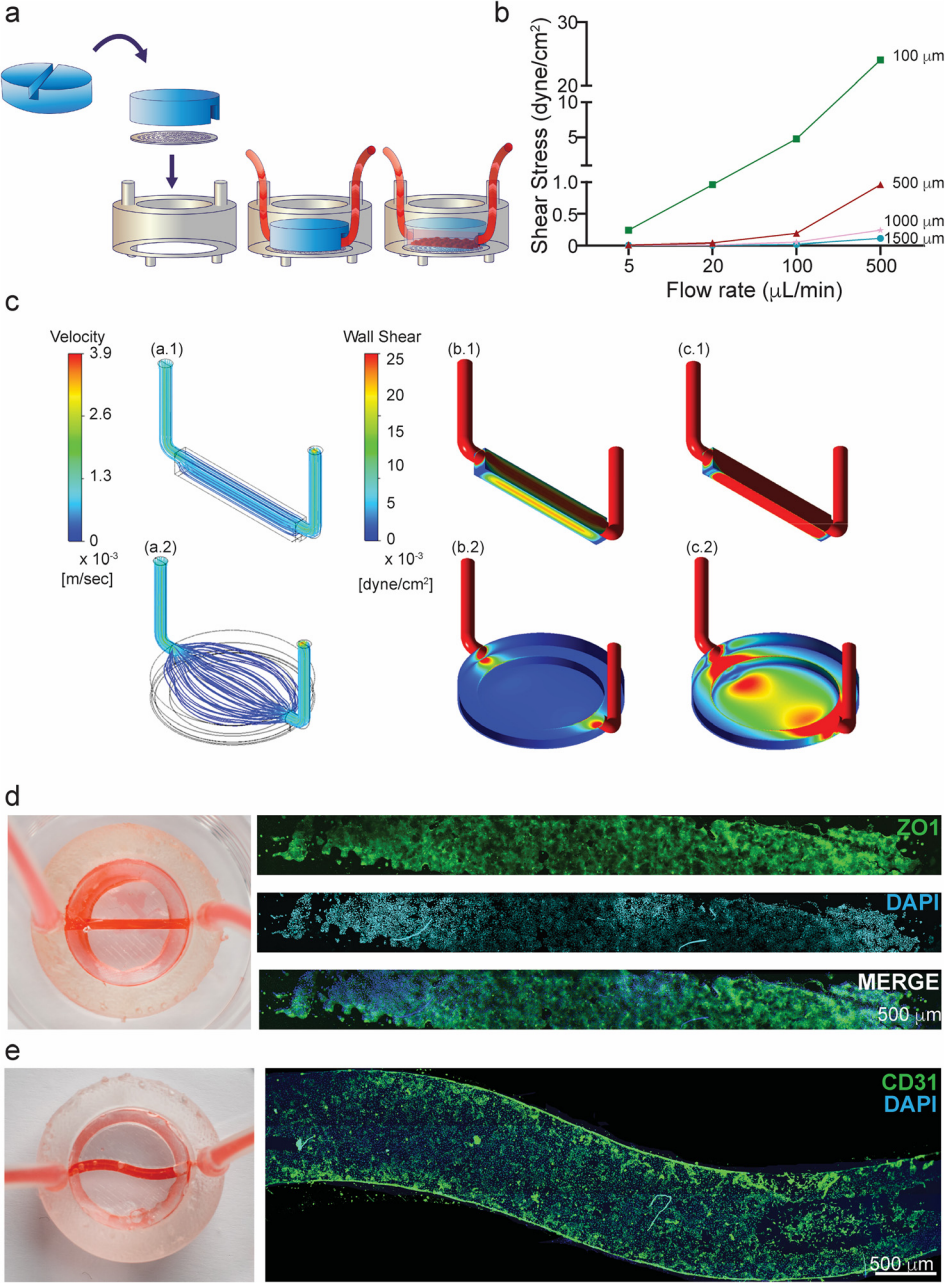
Linear and S-shaped reducers to control the flow. (a) Schematic experimental design of the linear insert-reducer that enables flow and shear stress to be controlled. (b) Plot showing shear stress values at different flow rate, changing the width of the reducer. (c) CFD calculated flow streamlines at a constant flow rate of 5 *μ*L/min through the chip with the reducer (a.1) and without (a.2). (b) CFD calculated WSS map at a constant flow rate of 5 *μ*L/min through the chip (b.1) with the reducer surface walls and membrane showing less than 0.025 dyne/cm^2^ on the membrane (max = 0.021 dyne/cm^2^). (b.2) WSS on the chip without reducer, showing less than 0.025 dyne/cm^2^ on the membrane (max = 0.0023 dyne/cm^2^). (c) CFD calculated WSS map at a constant flow rate of 50 *μ*L/min through the chip (c.1) WSS on the chip with the reducer, showing a significantly higher shear with 0.22 dyne/cm^2^ maximum shear. (c.2) WSS on the chip without the reducer, showing more than 0.025 dyne/cm^2^ on the walls (max = 0.027 dyne/cm^2^), which is higher than the WSS for the case with reducer and a flow rate of 5 *μ*L/min. (d) Photograph of the reducer integrated in the Insert-Chip and connected to an external pump with red color flushed inside. In the left panel, confocal tile scan reconstructions of Caco-2 cells grown under flow and able to form a confluent monolayer in the channel, immunostained for DAPI (blue) and ZO-1 (green). (e) Right panel; photograph of the S-shaped reducer. Left panel: HUVEC cells grown inside the channel and immunostained for CD-31 (green) and DAPI (blue).

Though the basic design of the Insert-Chip allows for the application of flow and the use of relatively small quantities of cells, we sought to take the design a step further: specifically, to enable the number of cells used to be further reduced, as well as to provide more precise control over the shear forces applied to the cells. To do so, we designed and fabricated a so-called reducer made of PDMS that easily can be placed in the chip [Fig. 7(a)], reducing the active surface area, and allowing channels to be created in any desired shape (Fig. 7). Moreover, by using the reducers and changing its width, it is possible to induce different shear stress, from 0.001 dyne/cm^2^ to almost 30 dyne/cm^2^, depending on the flow rate [see the plot in Fig. 7(b)]. Different flow profiles can be designed, combining Insert-chip with and without a reducer (Figs. S2 and S3) with the possibility to better control the flow and applied the desidered shear, even in case in which multiple Insert-Chip are connected. To better characterize the flow profile of the Insert-Chip, computational simulations were performed. The flow in the chip is laminar, producing parallel flow lines that wash the entire geometry with no visible flow separation and stagnant regions production a thoroughly perfused system in both the reduced [Fig. 7(c-a.1)] and the non-reduced [Fig. 7(c-a.2)] configurations. Although the system does develop a non-negligible Wall Shear Stress (WSS) at 5 *μ*L/min, it is significantly below any physiological shear stress that endothelial cells in the human body are normally exposed to.^46^

Nevertheless, the shear increases with the flow and can be brought to higher levels in both the reduced [Fig. 7(c-c.1)] and non-reduced configuration [Fig. 7(c-c.2)]. It can also be seen that in the reduced [Figs. 7(c-c.1) and 7(c-b.1)] configuration, the WSS is more uniform than in the non-reduced system [Figs. 7(c-c.2) and 7(c-b.2)], requiring the use of a reducer to produce uniform conditions.

In this work, we demonstrate two reducers, one with a linear shape [Fig. 7(d)] and one with an “S” shape [Fig. 7(e)]. Both reducers are constructed using a PDMS ring (17 mm length, 3 mm high) with channels in the desired formation integrated into the membrane (see Materials and Methods). The reducer enables the user to use just 20% of the whole membrane and thus to suffice with 15%–20% of the number of cells that would be needed for the basic version of the Insert-Chip, or for a regular well plate.

To demonstrate the use of the reducer, we cultured Caco-2 cells in the Insert-Chip with the linear shape reducer [Fig. 7(d)] and cultured HUVEC in the S shape reducer [Fig. 7(e)]. In order to achieve confluency, the cells were under a constant flow rate of 5 *μ*L/min, for 2 days. It can be seen that the Caco-2 [Fig. 7(d)] and the HUVEC [Fig. 7(e)] cells established adherens junctions in the epithelial and endothelial monolayer, indicating successful establishment of an intact barrier.

### Integrated TEER and MEA measurements

**FIG. 8.**
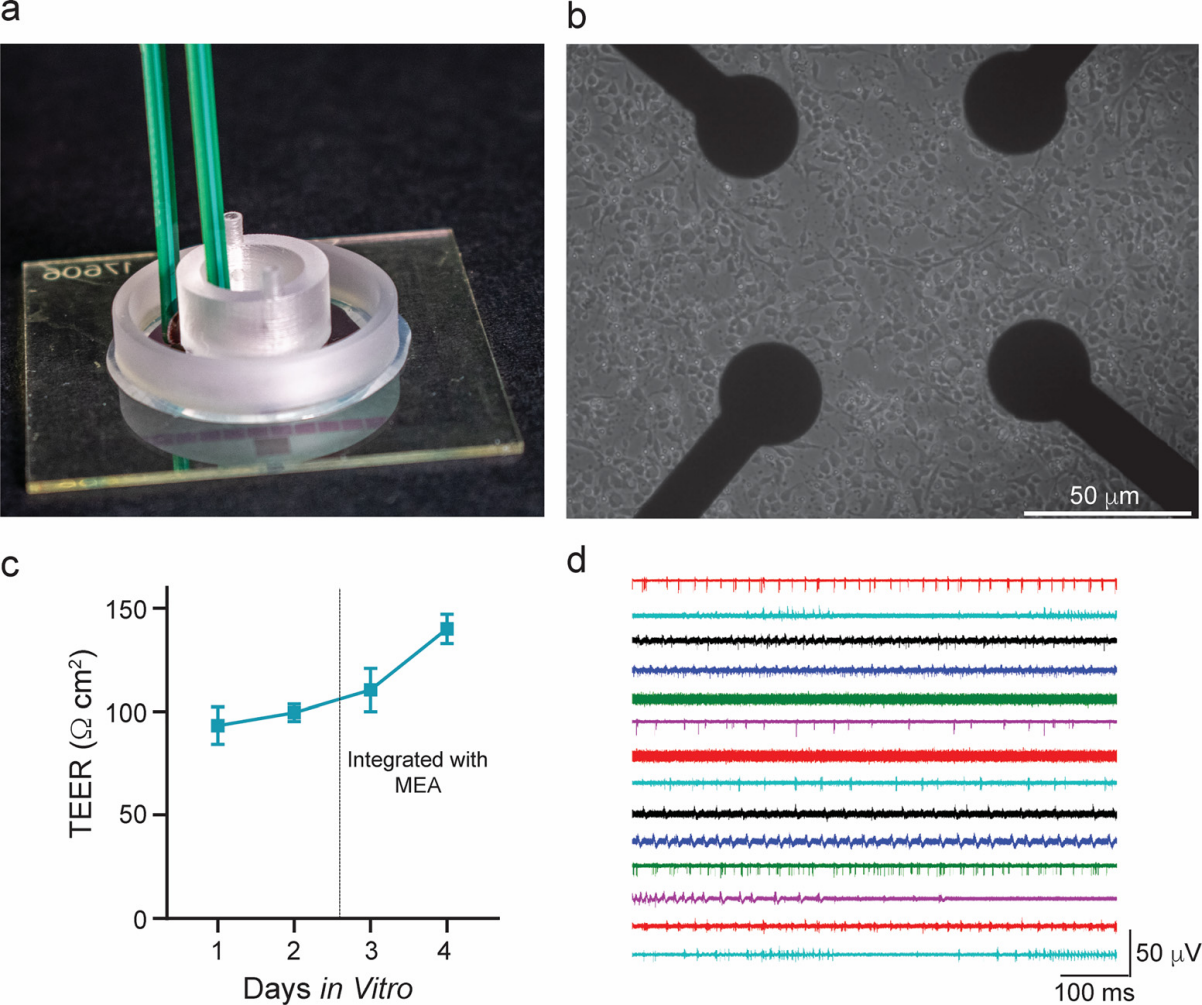
Integrating the Insert-Chip with MEA devices. (a) Photograph showing the Insert-Chip integrated in the MEA platform allowing for simultaneous TEER and electrophysiological measurements. (b) Rat hippocampal neurons cultured on the MEA device for 10–12 days *in vitro*. (c) TEER plot of HUVEC cultured on the Insert-Chip and integrated with the MEA. (d) Extracellular electrophysiological recordings of neuronal spontaneous activity recorded from 14 different electrodes after 10 days *in vitro*, simultaneously integrated with HUVEC grown on the Insert-Chip. Each color represents a different electrode.

As most of the parenchyma is surrounded by the barrier layer, there is a need for creating such co-culture systems, which allow to culture barrier layer and parenchymal, while assessing their functionality. We recently demonstrated an Organ-on-a-Chip with multiple sensors, in which it is possible to simultaneously measure both barrier function via TEER and the electrical activity of excitable cells, using MEAs.^32^ However, this platform requires custom fabrication and is therefore less accessible than commercial platforms. We designed the Insert-Chip to overcome this challenge; that is, it can be integrated into a commercial MEA platform (Fig. 8), such that permeability of the barrier tissue can be measured using a commercial TEER system, while the electrical activity of excitable cells is measured using the commercial MEA platform [Fig. 8(a)]. To demonstrate this capability, we used the neurovascular system as an example for such use. The blood brain barrier is a protective layer to the neurons, which is the brain parenchymal.^4,47–49^ HUVEC were cultured on the Insert-Chip; when the cells created a confluent monolayer, the chip was placed on top of a commercial MEA plate cultured with hippocampal neurons [Fig. 8(b)]. Barrier permeability was monitored with TEER [Fig. 8(c)], together with neuronal electrical activity [Fig. 8(d)], which remained robust over 10–12 DIV, giving the possibility to simultaneously monitor both cellular functionalities even if characterized by different maturation times.

It is important to note that such experiments are challenging to carry out with standard Organs-on-a-Chip, not only because of the technological aspect but also because of the biological aspect, which requires that both cell populations be at the same stages of maturation and functionality, which might be hard to coordinate. For example, it takes 1–3 days for the HUVEC to create a fully functional barrier; however, it takes at least 10 days to achieve robust neuronal activity. Use of the Insert-Chip enables the experimenter to culture each of the cell populations separately, and to combine them-by inserting the Insert-Chip into the MEA plate-only when both populations are mature.

## CONCLUSIONS

We have described the design, fabrication, and application of the Insert-Chip: an innovative yet straightforward Organ-on-a-Chip platform that can be easily fabricated (with 3D printing) integrated into standard cell culture systems. We demonstrated the Insert-Chip's capacity to grow two different types of cells (HUVEC and Caco-2-cells) under different flow patterns and to provide straightforward access to various types of measurements that are crucial in physiological and drug development studies, including barrier permeability. The modularity of the Insert-Chip, coupled with its capacity to enable multiple cell-types to be co-cultured and observed under flow conditions, will simplify experimental procedures that are currently highly complex in *in vitro* studies in academic and industry settings. In particular, the device has the potential to facilitate the study of cell–cell interactions, such as neurovascular coupling, essential to understanding the pathogenesis of multiple diseases.

## METHODS

### Insert-Chip development

*Insert-Chip design and fabrication.* The Insert-Chip was designed using SolidWorks CAD software (SolidWorks Corporation, MA). A schematic representation of the Insert-Chip fabrication can be visualized in Fig. 4. Prior to printing, model surfaces were checked, and a scaffold was added using PreForm software (PreForm 3.0.1, Formlabs, Inc.). Then, the chips were printed in a stereolithography Form2 3D printer (Formlabs, Somerville, Massachusetts), using a dental long-term (LT) clear resin (Formlabs), with unique mechanical and optical properties.^31^ After printing, the chips were washed in isopropyl alcohol (Avantor) in an ultrasound tank, to remove the unreacted resin, and then cured and dried in a UV curing system (Formlabs).

*Fabrication and assembly of additional components*. We used SolidWorks CAD software to design master-molds for fabrication of the device's additional components: the PDMS support ring, and two different “reducer” components aimed at reducing the active surface area in the chip and controlling the flow (a more advanced feature beyond the basic chip design; see Results and Discussion). The molds were printed with a commercial polylactic acid filament using a Raise 3D Pro2 Dual Extruder 3D Printer (Raise Technologies, Inc.). Prior to printing, model surfaces were checked, and, if needed, a scaffold was added using Idea Maker software (3.6.1, Raise Technologies, Inc.). Then, the molds were filled with PDMS prepared by mixing Sylgard 184^®^ (Dow Corning, Midland, MI) with the curing agent at a ratio of 1:10, followed by curing at 60 °C for almost 2 h. The resulting PDMS rings and reducers were cleaned in ethanol, dried at room temperature (RT), and then activated in oxygen plasma (Atto-BR-200-PCCE, Diener Electronic, Germany) for 30 s.

Polycarbonate (PC) membranes (0.4 *μ*m pore size, it4ip S.A., Belgium), 25 *μ*m thick, were cut to size with their protective backing on. The protective backings were then removed, and the PC membranes were rinsed with isopropanol, dried under a stream of compressed air, and activated in oxygen plasma for 2 min (Diener Electronic, Germany). Then, the membranes were immersed for 30 min in 5% aqueous solution of 3-aminopropyltriethoxysilane (APTES, Sigma-Aldrich) in order to introduce amino groups at the surface of the PC membrane.^32,33^ Then they were washed three times with water and dried under a stream of compressed air.

PDMS-rings or PDMS-reducers and PC membranes were then aligned and brought into contact, gently pressed together to ensure conformational contact, and baked at 60 °C.

The assembled parts were then inserted into the 3D-printed microfluidic Insert-Chip.

The ready-to-use assembled chip was sterilized using 70% ethanol for 30 min and was then washed with phosphate-buffered saline (PBS, Biological Industries) three times and sterilized under a UV lamp for 20 min.

*Validation of the flow gradient inside the chip*. Flow was controlled by an external peristaltic pump (IP-N 8, Ismatec, Cole-Parmer GmbH, Wertheim, Germany), and connections were in elastic tubing (inner diameter 1 mm, outer diameter 3 mm, Ismatec, Germany). The input tube was connected to the inlet of the chip, and the output tube was connected to a reservoir via the peristaltic pump.

*Cell culture*. To test the biocompatibility and the versatility of the Insert-Chip, we separately cultured epithelial and endothelial monolayers in Insert-Chips and monitored the cells under static and flow conditions. Furthermore, in order to demonstrate the significance of the Insert-Chip, cells were also cultured on commercially available Transwells (Corning). Moreover, to demonstrate how the Insert-Chip can be integrated into a more conventional cell culture environment, we cultured neuronal cells in MEAs, in which the Insert-Chip was subsequently placed.

*Epithelial culture*. For the epithelial model, we used human epithelial colorectal adenocarcinoma cells (Caco-2 cells, ATCC^®^ HBT-37^TM^, American Type Culture Collection, Rockville, MD). The passages of the Caco-2 cell line ranged from 26th to 40th. After thawing, the Caco-2 cells were cultured routinely in Dulbecco's Modified Eagle's Medium (DMEM, Biological Industries), supplemented with 10% heat-inactivated Fetal Bovine Serum (FBS, Biological Industries), 1% Glutamax (Gibco), and 1% Penicillin–Streptomycin–Amphotericin B (PSA, Biological Industries) solution, at 37 °C with 5% CO_2_ in a humidifying incubator. Cells were grown to 80%–90% confluence before being transferred inside the Insert-Chip. Before seeding, the porous membrane inside the Insert-Chip was treated with Matrigel Basement Membrane Matrix (Corning) used at 1:50 ratio with the culture medium, for 30 min in the incubator. The membrane was then rinsed with culture medium, and the Caco-2 cells, harvested with trypsin/EDTA solution (Biological Industries), were seeded at a density of 100 000 cells/cm^2^ and grown for 9–11 days, changing the medium every 4 days of cell culture.

For the flow condition, the tubing was sterilized by perfusing 70% ethanol throughout the system at a flow rate of 5 *μ*L/min for 2 h, to ensure a proper sterilization of the system. Following that, PBS was flushed into the entire system for an additional 2 h at the same flow rate, to ensure the complete removal of ethanol. Next, the solution containing Matrigel was flowed inside the Insert-Chip to coat the porous membrane, and the device was then incubated for 30 min. After incubation, the device was perfused with cell culture medium, and then the Caco-2 cells were seeded into the Insert-Chip. Next, the entire system was placed in the incubator, and the peristaltic pump was activated to perfuse culture medium at a constant flow rate of 5 *μ*L/min, for 2 days, to ensure the establishment of an intact monolayer of Caco-2 cells.

*Endothelial culture*. For the endothelial model, Human Umbilical Vein Endothelial cells (HUVEC, PromoCell GmbH, Heidelberg, Germany) were used. After thawing, the HUVEC were expanded in low-serum endothelial cell growth medium (PromoCell), at 37 °C with 5% CO_2_ in a humidifying incubator, and used at passage p3–p5. Cells were grown to 80%–90% confluence before being transferred inside the device. Before seeding, the PC membrane was treated with Entactin-Collagen IV-Laminin (ECL) Cell Attachment Matrix (Merck) diluted in DMEM (10 *μ*g/cm^2^), for 1 h in the incubator. Then, the HUVEC, harvested using a DetachKit (Promocell), were seeded inside the Insert-Chip at a density of 250 000 cells/cm^2^ and grown for 3–5 days. In the flow condition, the tubing was cleaned and sterilized as described above. Next, the solution containing ECL Matrix was flowed inside the chip and incubated for 1 h, and then cells were seeded. Then, the entire system was placed in the incubator, and the peristaltic pump was activated to perfuse culture medium at a constant flow rate of 5 *μ*L/min, overnight, to ensure the establishment of an intact monolayer of HUVEC.

*Cancer cells line*. To develop a tri-culture system, cancer cell lines (U87 glioblastoma and SH-SY5Y neuroblastoma cell lines, ATCC^®^) were used. After thawing, the U87 cells were cultured similarly to the epithelial cells and after reaching 80% confluency, they were seeded on the membrane. The SH-SY5Y cells were cultured in RPMI-F12 Medium (Biological Industries), supplemented with 10% FBS, 7.5% Sodium bicarbonate (Sigma-Aldrich), 1% Glutamax, and 1% Gentamycin (Gibco) solution, at 37 °C with 5% CO_2_ in a humidifying incubator. Cells were grown to 80%–90% confluence before being transferred inside the multi-well plate (Corning), after being harvested with trypsin/EDTA solution (Biological Industries).

*Neuronal culture*. Primary dissociated cultures were obtained from postnatal rats (p2–p3) as previously described.^34–36^ All experiments were approved by the local veterinary authority and the animal ethic committee of Tel Aviv university (approval ethic No. 01-19-079) and performed in accordance with Israeli law. All efforts were made to minimize animal suffering and to reduce the number of animals used. Neuronal hippocampal cells were plated on MEAs (Multi Channel Systems, Reutlingen, Germany) for network investigation. Prior to cell seeding, the MEA substrates were treated with polyethyleneimine (PEI, Sigma-Aldrich) in Borate buffer (Sigma-Aldrich) overnight at 4 °C. Then, the substrates were rinsed four times with distilled water, sterilized with UV for 1 h, and treated with laminin (20 *μ*g/mL, Sigma-Aldrich) diluted in plating medium containing Neurobasal Medium (Gibco), supplemented with FBS (5%, Biological Industries), B27 (2%, Gibco), Glutamax (1%, Gibco), and PSA (1%, Biological Industries), for 4 h, at 37 °C.

Neuronal hippocampal cells were then plated on coated MEA substrates in a plating medium and incubated at 37 °C in a humidified atmosphere enriched with 5% CO_2_. After 24 h had passed since seeding, the medium was replaced (80%) with serum-free neurobasal medium, supplemented with B27 (2%), Glutamax (1%), PSA (1%), and Gentamycin (1%, Gibco).^36,37^ Culture medium was renewed (50%) every 3 days from seeding. Plating was carried out at a nominal density of 70 000 cells/cm^2^. Cultures were then used for experiments after 9–12 days *in vitro* (DIV).

### Analytical studies

*Computational Fluid Dynamics (CFD) Model*. CFD simulations were conducted to characterize the flow in the chip and to determine the influence of the chip legs-height (LH) on the diffusion of mass. We derived the fluid volume from the chip geometries corresponding to the reduced and non-reduced configurations for the flow simulations, while a container was added in which the chip is submerged for the diffusion simulations. The geometries were meshed in ANSYS GAMBIT 19 R3 with the final elements number shown in Table I. All the simulations were conducted in ANSYS fluent 19 R3 using the constant laminar flow assumption for the flow simulations at two flow rates: 5 *μ*L/min and 50 *μ*L/min. The diffusion was modeled through the convection diffusion equation assuming constant diffusivity and mass production rate (see solved equations below). Since there are many configurations possible in the chip, we chose a simple configuration where the cells are located at the bottom of the reduced container producing CO_2_ at an arbitrary constant rate (0.0054 mmol/m^2^/s) while there are no cells anywhere else and there is no membrane. The CO_2_ diffusivity was taken^38^ to be 2.3 × 10^−9^ m^2^/s, and only one flow rate of 5 *μ*L/min was used. Finally, both steady state simulations to derive the final concentration gradients in the chips as well as transient simulations for 360-time steps of 1 s (6 min total) were performed to estimate the time scales involved and to produce movies of the diffusion process (see Movie S1 and S2).

**TABLE I.** Mesh types and number of elements.

Geometry	Element type	Number of elements
Non-reduced	Tetrahedral, prism	1M
Reduced	Tetrahedral, prism	300k
1 mm legs	Tetrahedral	400k
4 mm legs	Tetrahedral	400k

Solved equations:

Momentum:

$$\partial /\partial t\;(\rho \vec{v})+\nabla \cdot (\rho \vec{v}\;\vec{v})=-\nabla p+\nabla \cdot (\overset{¯¯}{\tau })+\rho \vec{g}+\vec{F}\;,$$ (1)

p—static pressure, $\rho \vec{g}$ and $\vec{F}$—gravitational body force and external body forces, respectively.

$$\overset{¯¯}{\tau }=\mu \left[\left(\nabla \vec{v}+{\vec{v}}^{T}\right)-\frac{2}{3}\nabla \cdot \vec{v}I\right],$$ (2)

μ—molecular viscosity, I—unit tensor, $2/3\;\nabla \cdot \vec{v}I$—volume dilation.

Continuity:

$$d\rho /\mathrm{dt}+\nabla \cdot (\rho \vec{v})=0.$$ (3)

Wall shear stress:

τ_w=μ ∂u/∂n, (4)

u—near wall velocity vector field. n—wall normal vector.

Convection diffusion

∂c/∂t=∇·(D∇c)−∇·(uc)+R, (5)

where c is concentration, D is the diffusivity coefficient in water, u is the velocity field obtained from Eq. (1), and R describes sources or sinks.

*Fixation, immunocytochemistry, and confocal imaging*. HUVEC, Caco-2, and the cancer cell lines were rinsed in PBS and fixed in 4% paraformaldehyde (PFA, Sigma-Aldrich) for 20 min at RT. Immunocytochemistry was carried out after permeabilization with 0.1% Triton X-100 (Sigma-Aldrich) in PBS for 10 min at RT and blocking for 30 min in FBS (5%) in PBS. Primary antibodies were applied overnight in PBS at 4 °C. The following primary antibodies were used for immunocytochemistry experiments: rabbit anti-ZO-1 (Abcam) and rabbit anti-CD-31 (Abcam), to stain the zona occludens-1 (a key component of tight junctions) in Caco-2 cells and the endothelial cell adhesion molecule 1 in HUVEC, respectively; mouse anti-GFAP (Abcam), to stain the Glial Fibrillary Protein in U87 cells; Phalloidin-iFluor 488 (Abcam), to stain actin in SY-SY5Y cells. Cells were then washed three times in PBS and stained with the secondary antibody for 1 h at RT. The secondary antibodies were anti-rabbit Alexa Fluor-488 (Invitrogen) and anti-mouse Alexa Fluor-594 (Invitrogen). After being washed four times with PBS, cells were mounted on a 0.17-mm-thick glass coverslip using DAPI-Fluoromount-G^®^ (SouthernBiotech), to stain the nuclei. Imaging was carried out using an inverted confocal microscope (Olympus FV3000-IX83), with appropriate filter cubes and equipped with 2×/0.08 NA, 10×/0.3 NA, 20×/0.8, and 60×/1.42 NA objectives. For imaging the entire channel within the PDMS-reducer, images were acquired by sequential tile scanning. Image reconstruction and processing were done using open-source ImageJ software.^39^

*Trans-epithelial endothelial electrical resistance (TEER)*. The barrier properties of the epithelial/endothelial monolayer were evaluated with TEER measurements along the cellular growth period. TEER was measured with the Millicell ERS-2 Voltohmmeter (Merck Millipore). TEER values (Ω cm^2^) were calculated and compared to those obtained in an Insert-Chip not containing cells, considered as blank, and were obtained from four different individual experiments, with two Insert-Chips used in each experiment.

*Permeability Assay*. HUVECs and Caco-2 were cultured on the Insert-Chip in static and under-flow condition. Permeability of the monolayer was assessed by measuring leakage of Fluorescein isothiocyanate (FITC)-dextran (Sigma-Aldrich) administered to the upper compartment of the Insert-Chip at different time points. One hour after adding dextran, the fluorescence intensity of the medium in the lower compartment was measured by a fluorescent plate reader (Multiskan Go, Thermo Scientific), at an excitation of 492 nm and emission of 518 nm (2 Insert-Chip for each condition).

*MEA recording*. Neuronal network extracellular recordings were carried out using the MEA60 system (Multi Channel Systems). Primary hippocampal cultures were plated on Titanium Nitride (TiN) MEAs with 60 electrodes (30 *μ*m dimeter, 200 *μ*m inter-electrode spacing). Raw data were monitored and recorded by using the commercial software MCRack (Multi Channel Systems), at 37 °C, in the presence of cell culture medium. The recorded events were analyzed offline with NeuroExplorer 5.127 software (Nex Technologies, Colorado).

*Statistical analysis*. The results are presented as the mean ± SD. Statistically significant differences among multiple groups were evaluated by two-way analysis of variance, followed by the Holm–Sidak test for multiple comparison (GraphPad Prism 8.4.3). A statistically significant difference between two data sets was assessed and P < 0.05 was considered statistically significant.

## SUPPLEMENTARY MATERIAL

See the supplementary material for demonstration of the Insert-Chip to support tri-culture system (Fig. 1), schematic of flow diagrams in the Insert-Chip without the reducer (Fig. 2), schematic of flow diagrams in the Insert-Chip with the reducer (Fig. 3), simulation how the height of Insert-Chip affects the CO_2_ concentration (Movie 1), 3D flow simulations (Movie 2), and dye diffusion over time for observing the chip properties (Movie 3).

## AUTHORS’ CONTRIBUTIONS

R.R. performed all fabrication, cell biology, and confocal experiments. A.E. contributed to the chip design and fabrication. B.L.R. assisted with the photos. Y.K., M.E., and N.K. performed the flow simulations. R.R. and B.M.M. conceived the study and the experimental design and wrote the manuscript.

## Data Availability

The data that support the findings of this study are available from the corresponding author upon reasonable request.
